# *Acetobacteraceae* as exopolysaccharide producers*:* Current state of knowledge and further perspectives

**DOI:** 10.3389/fbioe.2023.1166618

**Published:** 2023-03-30

**Authors:** Julia Wünsche, Jochen Schmid

**Affiliations:** Institute for Molecular Microbiology and Biotechnology, University of Münster, Münster, Germany

**Keywords:** bacterial exopolysaccharides, *Acetobacteraceae*, acetan-like biopolymers, bacterial cellulose, levan, xanthan-like biopolymers

## Abstract

Exopolysaccharides formation against harmful biotic and abiotic environmental influences is common among bacteria. By using renewable resources as a substrate, exopolysaccharides represent a sustainable alternative to fossil-based polymers as rheological modifiers in food, cosmetics, and pharmaceutical applications. The family of *Acetobacteraceae,* traditionally associated with fermented food products, has demonstrated their ability to produce a wide range of structural and functional different polymers with interesting physicochemical properties. Several strains are well known for their production of homopolysaccharides of high industrial importance, such as levan and bacterial cellulose. Moreover, some *Acetobacteraceae* are able to form acetan-like heteropolysaccharides with a high structural resemblance to xanthan. This mini review summarizes the current knowledge and recent trends in both homo- and heteropolysaccharide production by *Acetobacteraceae*.

## 1 Introduction

The biosynthesis of carbohydrate polymers is a common characteristic of both prokaryotic and eukaryotic organisms. Extracellularly secreted glycosides are classified as exopolysaccharides (EPS). Major functions include the protection against environmental influences such as desiccation, osmotic stress, phagocytosis, or antibiotics. Furthermore, intercellular interactions like cell recognition and surface adhesion are also promoted (Suresh Kumar et al., 2007; Corbett et al., 2010; Moradali and Rehm, 2020). EPS are known for their high diversity in terms of physicochemical and rheological properties (Hundschell and Wagemans, 2019).

EPS are either classified as homopolysaccharides or heteropolysaccharides based on their general chemical complexity. Although homopolysaccharides consist per definition of only one kind of monomer, the linkage pattern usually varies a lot resulting in branched (e.g. glycogen) and unbranched (e.g. cellulose) polymer structures. Heteropolysaccharides, on the other hand tend to have highly complex structures as they are composed of at least two different sugar moieties. Additionally, polymers can be further decorated with organic and inorganic moieties such as acetyl, pyruvyl, glyceryl, succinyl, and sulphate constituents (Sutherland, 1990; Freitas et al., 2011; Nwodo et al., 2012). Along with the conformation of glycosidic linkages, a vast amount of potential structures emerge, giving rise to a wide range of physicochemical properties (Freitas et al., 2011).

Those variable material properties in combination with a high natural water-binding capacity are one of the main reasons for the broad commercial potential of EPS, representing an alternative to replace petrochemical polymers in current applications (Kamaruddin et al., 2021). However, only a rather limited number of microbial EPS can be regarded as industrially established, e.g. hyaluronan, xanthan, pullulan, dextran and gellan gum (Sutherland, 1998; Heinze et al., 2006; Osmałek et al., 2014; Singhsa et al., 2018; Schilling et al., 2020; Meliawati et al., 2022). Low titers and yields, as well as expensive downstream processing, result in high production costs and consequently impede the industrial establishment of new strains and polymers. Thus, EPS are up to now mainly used in high-value niche products in cosmetics, food, and pharmacy (More et al., 2021). This applies in particular to the Gram-negative *Acetobacteraceae* which are mainly known for the production of fermented food products like vinegar, kefir, or acetic acid production but have also demonstrated their ability to produce structurally different EPS with interesting physicochemical properties (La China et al., 2018).

This mini review aims to summarize the knowledge of homopolysaccharides and heteropolysaccharides production in *Acetobacteraceae* with regard to the current state of strain development, bioprocess optimization, and knowledge of rheological properties to evaluate the *status quo* and provide a further outlook on this particular group of promising biopolymers. Since the phylogenetic classification of *Acetobacteraceae* is not finalized and currently consists of 47 genera in April 2022, the classification of the original publication is used in this article (Parr et al., 2014).

## 2 Homopolysaccharide production in *Acetobacteraceae*

### 2.1 Levan

*Acetobacteraceae* are known for their production of high-value homopolysaccharides such as levan. Levan synthesis is widespread within the family of *Acetobacteraceae* and was reported for numerous organisms including the genera *Neoasaia*, *Kozakia*, and *Gluconobacter* (Kornmann et al., 2003; Hermann et al., 2015; Hövels et al., 2020; Anguluri et al., 2022). Its formation is catalyzed by an extracellular enzyme named levansucrase (LS, EC 2.4.1.10). By cleaving sucrose, LSs are capable of polymerizing the emerging D-fructose monomers to β-(2,6) linked polyfructans (Öner et al., 2016; Xu et al., 2019). Meanwhile, D-glucose as a sacrificial substrate is metabolized and used for bacterial growth resulting in a theoretical maximum levan yield of 0.5 g_Lev_/g_Suc_. Based on the evaluation of phylogenetic clades in *Acetobacteraceae*, two types of LS with different ecological relationship could be distinguished, differing in yield and molecular weight (Jakob et al., 2019). Levan is currently highly requested as a stabilizer, emulsifier, and flavor enhancing agent in food applications (Öner et al., 2016).

Up to now, investigations on levan production by *Acetobacteraceae* focused mainly on the characterization of wild-type strains which might be explained by the large number of levan-producing strains in this particular family as well as high titers already obtained under non-optimized cultivation conditions (Table 1). Comparatively low titers of 6.3 g L^-1^ and 7.3 g L^-1^ were reported for cultivations in shake flask experiments for *Gluconobacter cerinus* DSM 9533 and *Neoasaia chiangmaiensis* NBRC 101099, respectively. Slightly higher titers of 7.8 g L^-1^ were obtained under identical conditions for *Kozakia baliensis* DSM 14400 (Jakob et al., 2012). However, for all strains carbon yields remained at a low level of approximately 0.1 g_Lev_/g_Suc_. Anguluri et al. (2022) reported a final titer of 35.0 g L^-1^ for the same *Neoasaia chiangmaiensis* strain after increasing the final sucrose concentration up to 250 g L^-1^. Despite increased product titers, in both studies carbon yields of only 0.10 and 0.14 g_Lev_/g_Suc_ were achieved, respectively. For *Gluconacetobacter diazotrophicus* PA1 5 a decent titer of 24.8 g L^-1^ was obtained showing similar carbon yields (0.16 g_Lev_/g_Suc_). Significantly higher yields of 0.33 and 0.38 g_Lev_/g_Suc_ were observed for the species *Acetobacter xylinum* NCIM 2526 and *Gluconobacter frateurii* TMW 2.767, respectively (Jakob et al., 2012; Semjonovs et al., 2016). Recently, *Tanticharoenia sakaeratensis* TBRC 22 was identified as a promising alternative production strain with a final levan titer of 24.7 g L^-1^ using 200 g L^-1^ sucrose as the initial substrate concentration (Aramsangtienchai et al., 2020). By plasmid-based overexpression of the native LS gene *sacB* in *Gluconobacter japonicus* LMG 2417, LS activity could be successfully increased 2.5-fold compared to the wild-type strain, resulting in higher space-time yields and titers (Hövels et al., 2020). In general, in-depth investigations on bioprocess optimization approaches for levan production in *Acetobacteraceae* seem to be rare and mainly limited to the identification of the best media compositions so far as extensively reviewed by Öner et al. (2016).

**TABLE 1:** Overview of homopolysaccharides producing *Acetobacteraceae*.

EPS	Strain	Titer [g∙L^-1^]	Yield [g_EPS_/g_Sub_]	Cultivation conditions in the selected study [g∙L^-1^]	Reference
Levan	*Gluconobacter cerinus* DSM 9533	6.3	0.08	20 sodium gluconate, 3 yeast extract, 2 peptone, 3 glycerol, 10 mannitol, 80 sucrose, pH 6.0	Jakob et al., 2012
				50 mL cultivation volume in shake flasks, 30°C, 24 h, 180 U min^-1^	
Levan	*Neoasaia chiangmaiensis* NBRC 101099	7.3	0.09	20 sodium gluconate, 3 yeast extract, 2 peptone, 3 glycerol, 10 mannitol, 80 sucrose, pH 6.0	Jakob et al., 2012
				50 mL cultivation volume in shake flasks, 30°C, 24 h, 180 U min^-1^	
		35.0	0.14	250 sucrose, 0.5 yeast extract, 0.5 polypeptone, 0.73 Na_2_HPO_4_, 0.115 citric acid, 0.05 MgSO_4_ (%, *w/v*)	Anguluri et al., 2022
				40 mL cultivation volume in shake flasks, 30°C, 140–200 rpm
Levan	*Kozakia baliensis* DSM 14400	7.8	0.10	20 sodium gluconate, 3 yeast extract, 2 peptone, 3 glycerol, 10 mannitol, 80 sucrose, pH 6.0	Jakob et al. (2012)
				50 mL cultivation volume in shake flasks, 30°C, 24 h, 180 U min^-1^	
Levan	*Gluconobacter cerinus* DSM 9533	56.7	0.23	250 sucrose, 0.5 yeast extract, 0.5 polypeptone, 0.73 Na_2_HPO_4_, 0.115 citric acid, 0.05 MgSO4 (%, *w/v*)	Anguluri et al. (2022)
				40 mL cultivation volume in shake flasks, 30°C, 140–200 rpm	
Levan	*Gluconobacter frateurii* TMW 2.676	30.0	0.38	20 sodium gluconate, 3 yeast extract, 2 peptone, 3 glycerol, 10 mannitol, 80 sucrose, pH 6.0	Jakob et al. (2012)
				50 mL cultivation volume in shake flasks, 30°C, 24 h, 180 U min^-1^	
Levan	*Tanticharoenia sakaeratensis* TBRC22	24.7	0.25	5 peptone, 5 NaCl, 1.5 meat extract, 1.5 yeast extract, 200 sucrose	Aramsangtienchai et al. (2020)
				5% bacterial culture, 37°C, 60 h, 180 rpm	
Levan	*Gluconacetobacter diazotrophicus* PA1 5	24.8	0.17	LGIM media with 150 sucrose, supplemented either with 3 (NH_4_)_2_SO_4_ or 1.5 tryptone/yeast extract	Stephan et al. (1991), Molinari and Boiardi (2013)
				1.5 L working volume fermentation, 30°C, 15–20 L h^-1^, pH 6.0	
Levan	*Acetobacter xylinum* NCIM 2526	13.2	0.33	40 sucrose, 20 bacteriological peptone, 1.0 (NH_4_)_2_SO_4_, 1.0 KH_2_PO_4_, 1.0 MgSO_4_∙7H_2_O	Srikanth et al. (2015)
				28°C, 60 h	
BC	*Gluconacetobacter sp.* RKY5	5.52	0.37	15.0 glycerol, 8.0 yeast extract, 3.0 KH_2_PO_4_, 3.0 acetic acid	Kim et al. (2007)
				1 L working volume in a rotary biofilm conductor, 30°C, 96 h, 15–35 rpm	
BC	*Gluconacetobacter intermedius* SNT-1	12.6	0.63	20 glucose, 5 yeast extract, 5 polypeptone, 2.75 Na_2_HPO_4_, 1.15 citric acid monohydrate, pH 6.0	Tyagi and Suresh (2016)
				Static conditions, 30°C, 120 h	
BC	*Gluconacetobacter xylinus* PTCC 1734	1.8	0.03	Hestrin-Schramm, Yamanaka or Zhou media with either date syrup, glucose, mannitol, sucrose, or (food-grade) sucrose	Mohammadkazemi et al., (2015)
				28°C, 168 h, 150 rpm	
		1.9	0.01	20 carbon source (glycerol, sucrose, mannitol, fructose), 5 peptone, 5 yeast extract, 2.7 Na_2_HPO_4_, 1.15 citric acid	Jalili Tabaii and Emtiazi, (2015)
				30 mL working volume, static cultivation, 28°C, 480 h, pH 6.0	
BC	*Gluconacetobacter xylinus* ATCC 23770	10.8	n.a.	Cotton-based waste textiles, 2.5 D-mannitol, 0.5 yeast extract, 0.3 peptone, pH 5.0 (%, *w/v*)	Hong et al., 2012
		8.3	0.66	12 wheat straw hydrolysate, 0.3 peptone, 0.5 yeast extract (%, *w/v*)	Chen et al., (2013)
				Static cultivation, 30 °C, 168 h	
BC	*Gluconacetobacter xylinus* NRRL B-42	10.0	0.5	2.0 glycerol/cane molasses, 0.5 peptone, 0.5 yeast extract, 0.27 disodium phosphate, 0.115 citric acid (%, *w/v*), pH 6.0	Vazquez et al. (2013)
				Static cultivation, 5:1 (volume flask: volume media), 28°C, 336 h, pH 5.0	
BC	*Komagataeibacter medellinensis*	3.3	0.17	1/2/3 carbon source (glucose, sucrose, fructose), 0.5 yeast extract, 0.5 peptone, 0.5 Na_2_HPO_4_, 0.267 citric acid (%, *w/v*)	Molina-Ramírez et al. (2017)
				100 mL working volume, static cultivation, 192 h, pH 6.0	

Levan formation is controlled by LS as the only enzyme in the biosynthesis process (Schmid, 2018). Depending on the available fructosyl acceptor molecule, the enzyme catalyzes hydrolysis, transfructosylation (in the presence of small oligosaccharides) and polymerization (in the presence of a increasing fructan chain) (Li et al., 2015). In consequence, defined process conditions are essential to push the reaction equilibrium towards levan formation while avoiding product degradation. Several studies in Gram-positive and Gram-negative bacteria demonstrated the importance of the right temperature settings during cultivation and the influence of metal ions, which need to be carefully determined for each LS respectively (Park et al., 2003; Rairakhwada et al., 2010; Tian et al., 2011; Belghith et al., 2012). Moreover, the optimal length of the fermentation process has to be carefully evaluated since the equilibrium naturally tends towards hydrolysis with the depletion of sucrose as the substrate during the cultivation (Chambert et al., 1974; Hernandez et al., 1995). In a recent study of Anguluri et al. (2022), the authors could show that a longer process time of 96 h resulted in a significant product decrease for *Kozakia baliensis* DSM 14400 in comparison to 48 h of cultivation. In contrast, 96 h of fermentation increased yields for *Neoasaia chiangmaiensis* NBRC 101099 by 32 %, thus underlining the need for further strain-specific bioprocess optimization approaches.

### 2.2 Bacterial cellulose

In addition, *Acetobacteraceae* are associated with the biosynthesis of β-(1,4) linked polyglucans which are referred to as bacterial cellulose (BC). Due to the absence of hemicellulose and lignin as present in its eukaryotic plant counterpart, BC is known to be of extremely high purity. Moreover, due to the lack of required energy-intensive downstream processing which is essential for plant-derived cellulose, BC typically demonstrates a low amount of inorganic impurities (Klemm et al., 2011). In applications, BC is valued for its high crystallinity and superior mechanical strength (Nakayama et al., 2004; Castro et al., 2011; Ul-Islam et al., 2012). All of these properties are highly desired in current product development and makes BC an excellent biocompatible material for pharmaceutical products. High potential is reported for wound dressing materials, drug delivery systems and packing materials (Czaja et al., 2006; Abeer et al., 2014; El-Gendi et al., 2023). In order to address this trend, current research focus on *in situ* (optimization during fermentation) and *ex situ* (optimization of existing microfibers) BC properties modifications (Stumpf et al., 2018; Cazón and Vázquez, 2021). Addition of 30% (*v/v*) aloe vera gel for instant resulted in significantly increased mechanical strength and water absorption capacity (Saibuatong and Phisalaphong, 2010).

Traditionally, BC is generated in the air-liquid interface in static fermentation processes. By accumulation on the surface, a gelatinous layer around bacterial cells is formed (Cannon and Anderson, 1991; Jonas and Farah, 1998). In consequence, maximum yields positively correlate to the surface area (Masaoka et al., 1993). However, this leads to several practical problems during production, e.g. insufficient oxygen supply and long lasting fermentations, or a barely separable mixture of biomass and polymer (Shoda and Sugano, 2005; Hsieh et al., 2016). Especially when it comes to industrial scale-up, these issues limit the economic feasibility. Production in large scale are therefore conducted in modified horizontal lift, gas lift, rotary discs and membrane bioreactors (Shi et al., 2014). Titers of 6.2 g L^-1^ BC were achieved by using a rotary biofilm conductor with eight discs (Kim et al., 2007). However, it has to be mentioned that the optimal static process conditions are often not met completely in those set-ups.

In order to reduce manufacturing costs, optimization approaches focus nowadays more on the establishment of low-cost media and the investigation of alternative raw materials in order to replace glucose, fructose or glycerol as established substrates (Jalili Tabaii and Emtiazi, 2015; Mohammadkazemi et al., 2015; Molina-Ramírez et al., 2017; Revin et al., 2018; Saleh et al., 2021; El-Gendi et al., 2022). Tyagi and Suresh achieved remarkable titers of 12.6 g L^-1^ for BC with *Gluconaceteobacter intermedius* SNT1 on sugarcane molasses (Tyagi and Suresh, 2016). Numerous further publications indicate the high potential of this approach, including the redirection of waste streams and by-products of chemical processes (Hong et al., 2012; Chen et al., 2013; Vazquez et al., 2013; Barshan et al., 2019). In addition, BC can also be produced in submerge cultivation systems through agitated or aerated bioreactors with respectable titers between 15 and 20 g L^-1^ BC (Kouda et al., 1998). However, the occurrence of unintended cellulose-deficient mutants and therefore a decline in product titers have been reported in several studies (Vandamme et al., 1998; Jung et al., 2005; Matsutani et al., 2015). Moreover, higher oxygen supply during cultivation was demonstrated to alter BC morphology towards granule and pellet formation, thus affecting material properties (Singhsa et al., 2018). Recent trends also focus on the impact of additives and co-cultivations in order to optimize both BC titers and rheological properties. Positive effects were demonstrated for pullulan, whose supplementation resulted in improved mechanical polymer properties and 4.4-fold increased BC yield (Hu et al., 2022).

Contrarily to the previously discussed levan-type polyfructans, BC biosynthesis and polymerization is more complex as it is organized in a cellulase synthase operon consisting of at least four different genes (Römling and Galperin, 2015). Several studies aimed to increase and optimize BC production on a molecular level. In order to enable metabolization of sucrose as a cheaper carbon source, a recombinant sucrose synthase was successfully expressed in *Acetobacter xylinum* BRP 2001. By this, final titers on glucose as carbon source could be doubled to 8 g L^-1^ (Nakai et al., 1999). Furthermore, 28-fold increased BC formation was demonstrated for *Acetobacter xylinus* ITZ3 after the successful genomic integration of the β-galactosidase *lacZ*, thus adding lactose to the group of potential substrates (Battad-Bernardo et al., 2004). Heterologous expression studies might present one way to overcome the prominent issue of long lasting cultivation by *Komagataeibacter* spp. Imai et al. (2014) demonstrated BC production *via* the much faster growing *Escherichia coli* by heterologous expression of the cellulase synthase complex subunits *cesAB* as well as the cyclic-di-GMP diguanylate cylase *dgc* of *Gluconacetobacter xylinus* (Imai et al., 2014). Recently, for the first time, a CRISPR-Cas tool was successfully applied in *Komagataeibacter* spp. The study of Huang et al. (2020), used a CRISPRi-based approach to downregulate *galU*, which controls the metabolic flux between the BC synthesis and the pentose phosphate pathway. By minimizing the expression level of *galU*, BC of higher crystallinity was obtained, although enhanced material porosity as an severe adverse effect was documented as well (Huang et al., 2020).

## 3 Heteropolysaccharide production in *Acetobacteraceae*

**FIGURE 1:**
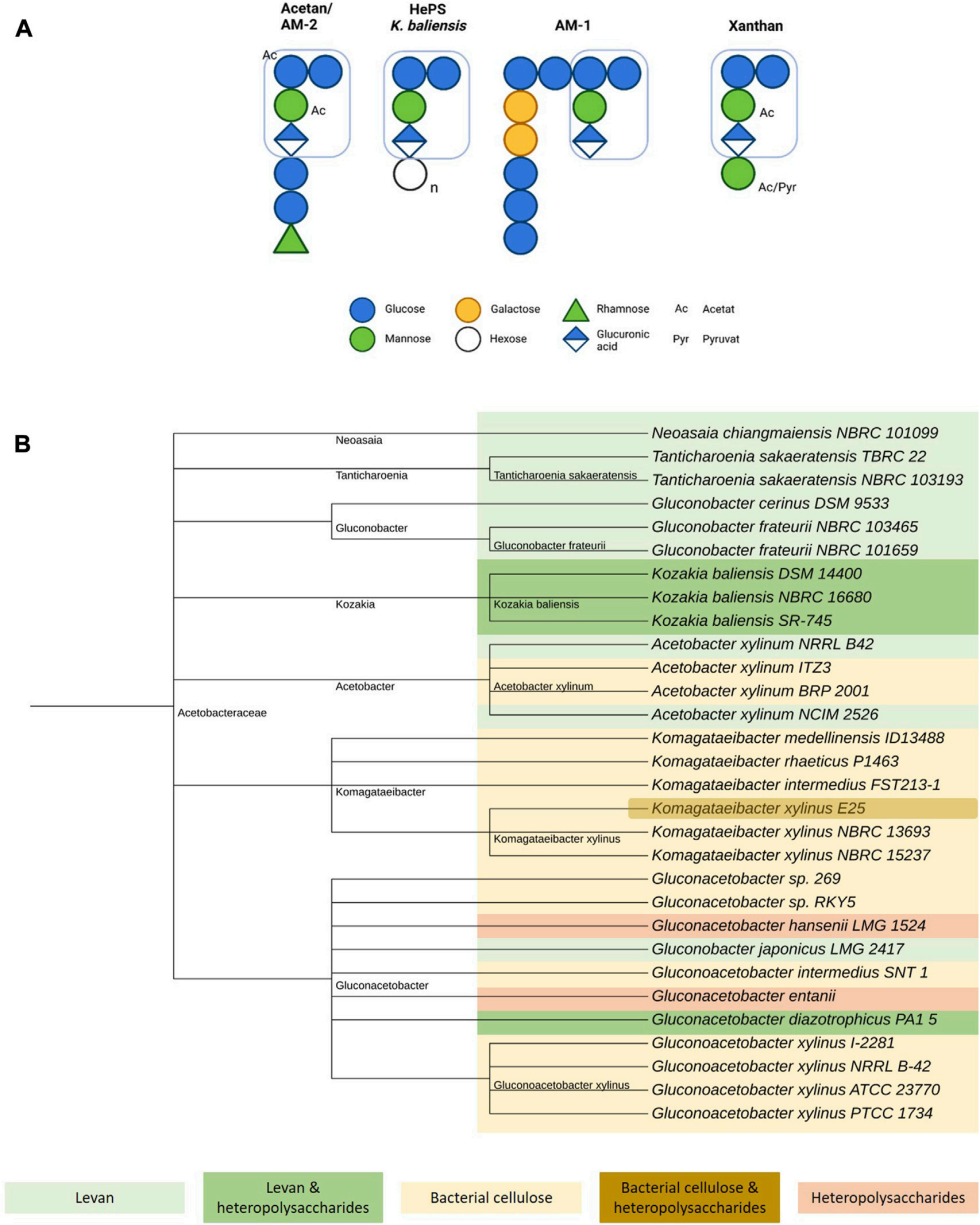
Overview of acetan-like heteropolysaccharide production in *Acetobacteraceae*. **(A)** Schematic comparison of selected acetan-like heteropolysaccharides produced by *Acetobacteraceae* including acetan, a yet unnamed heteropolysaccharide by *Kozakia baliensis*, AM-1, and xanthan (Jansson et al., 1975; Tayama et al., 1985, 1986; Edwards et al., 1999; Brandt et al., 2018). The xanthan-like core structure is marked for all polymers. Figure created with BioRender.com. **(B)** Taxonomy tree of EPS-producing *Acetobacteraceae.* Levan producing strains are marked in light green, levan and acetan-like heteropolysaccharides producing strains in dark green, bacterial cellulose producing strains in yellow, bacterial cellulose and acetan-like heteropolysaccharide producing strains in brown and only acetan-like heteropolysaccharide producing strains in light red. Figure created with iTOL (Letunic and Bork, 2021).

The formation of heteropolysaccharides within the family of *Acetobacteraceae* has been investigated in several publications (Brandt et al., 2016; Škraban et al., 2018; Rath et al., 2022). Interestingly, many if not all of the yet elucidated heteropolysaccharides in this family are structural related to acetan, whose production was first described in *Acetobacter xylinum* (Figure 1). Acetan consists of a molar subunit ratio of 4 : 1: 1 : 1 (glucose, mannose, glucuronic acid, rhamnose). In addition to the cellulose-like backbone with a trisaccharide branching sidechain at every other glucose monomer, the first two monomers of the side chain, identified as mannose and glucuronic acid, are identical in sequence and linkage pattern to the core structure of xanthan gum (Jansson et al., 1975; Couso et al., 1987). However, the further side chain composition and acetyl- and pyruvation pattern differs, giving rise to variety of structures and different rheological properties (Tayama et al., 1986; Brandt et al., 2018; Rath et al., 2022). This resemblance is also displayed by a high degree of homology between the heteropolysaccharides encoding genomic regions in *Acetobacteraceae* and the xanthan biosynthesis cluster of *Xanthomonas campestris* (Becker et al., 1998). Genetic alignments demonstrated a strong homology for *aceA* of *Acetobacter xylinum* and *gumD* from *Xanthomonas campestris*, both of these so-called priming glycosyltransferases in heteropolysaccharides synthesis initiating the assembly of the repeating unit at an undecaprenyl-pyrophosphate lipid anchor (Griffin et al., 1994; Schmid and Sieber, 2015). Moreover, a more recent study of Brandt et al. (2016) compared and confirmed homologies in the underlying heteropolysaccharides biosynthesis clusters of *Kozakia baliensis* DSM 14400 and NBRC 16680, *Gluconacetobacter diazotrophicus* PA1 5, *Komagataeibacter xylinus* E25 and *Xanthomonas campestris* ATCC 33913. Although all of the examined clusters showed high structural similarities, variations in numbers and size of the predicted genes and clusters were revealed, explaining the strain-dependent differences in the resulting polymer structures.

Xanthan gum is highly requested in industrial applications as a viscosifier due to its pseudoplastic behavior, high salt tolerance and thermostability amongst others properties (Chaturvedi et al., 2021). Similar beneficial rheological characteristics have also been described for the structure-related heteropolysaccharides of *Acetobacteraceae*, although studies in this field are rather limited. Already in 1989, the first rheological characterization of acetan was performed (Morris et al., 1989). Moreover, rheological behavior investigations of heteropolysaccharides produced by *Kozakia baliensis* confirmed pseudoplastic behavior and high viscosity (Brandt et al., 2018). Although the first results appear to be promising, further in-depth rheological studies are absolutely required in consideration of the rather insufficient data situation.

With regard to strain cultivation, respectable titers for heteropolysaccharides production in *Acetobacteraceae* wild-type strains have been reported. A titer of 5.4 g L^-1^ acetan was obtained under controlled cultivation for *Gluconoacetobacter entanii* (Velasco-Bedrán and López-Isunza, 2007). Significantly higher titers of 11.3 g L^-1^ gluconacetan were achieved for *Gluconoacetobacter xylinus* I-2281, likewise under controlled fermentation conditions in bioreactors and using fructose as the main carbon source (Kornmann et al., 2003). In a recent study based on an systematic optimization by use of experimental design, the putative gluconacetan titer for *Gluconoacetobacter* sp. could be even increased to 25.4 g L^-1^ although the parallel formation of second ribose-containing heteropolysaccharides could not be completely precluded (Rath et al., 2022). By using glycerol as the carbon source, the authors aimed to minimize the formation and accumulation of undesirable oxidized compounds such as gluconates, which affect the pH of the fermentation broth and contaminate the final polymer. The oxidation of sugar and alcohols within the respiratory chain mechanism in the outer membrane is a characteristic feature of *Acetobacteraceae* (Adachi and Yakushi, 2016). As the formation of numerous (by-) products is a main issue for *Acetobacteraceae*, the right choice of carbon source and cultivation conditions are critical for EPS production and should be investigated further. Moreover, cultivation of *Gluconacetobacter hansenii* LMG 1524 in a media consisting of glycerol as the main carbon source and ammonium sulphate as the corresponding nitrogen source resulted in a maximum titer of 1.22 g L^-1^, in comparison to other examined carbon and nitrogen sources variations (Valepyn et al., 2012). This once more underlines the importance of strain-dependent bioprocess optimization as the authors were also able to demonstrate that lower temperatures at 25°C and a slightly decreased pH value of 5.0 favored EPS over cell biomass production. Cultivation in the presence of two initial carbon sources (glucose and fructose) and 200 mg L^-1^ of magnesium resulted in a titer of 3.9 g L^-1^ for *Kozakia baliensis* NBRC 16680 in shake flasks (Brandt et al., 2018). Additional magnesium has previously been shown to positively affect heteropolysaccharides production in *Pseudomonadaceae* (Vargas-García et al., 2001). However, in the previously mentioned study of Brandt, significantly increased EPS production in *Kozakia baliensis* due to the presence of magnesium could not be confirmed.

## 4 Conclusion and further perspectives

The increasing demand for healthier and more sustainable products as driven by the customers, offers a unique chance to increase the replacement of petrol-based compounds and chemicals in a broad range of applications. Hugh potential can be assumed for EPS which possess the required material properties for usage in food, cosmetic and pharmaceutical applications. This applies especially to EPS produced by *Acetobacteraceae*, whose homopolysaccharides levan and BC have shown promising material properties. Due to their structural resemblance to xanthan, acetan-like heteropolysaccharides are also highly interesting.

However, for industrial scale-up processes and in order to enhance economic feasible production, future research must address the need for higher titers and carbon yields as well as utilization of second-generation feed stocks to produce both homopolysaccharides and heteropolysaccharides. In addition, investigation and improvement of rheological polymer properties *via* genetic engineering or fine-tuned formulations are also highly desired to promote future application development for acetan-like polymers.
